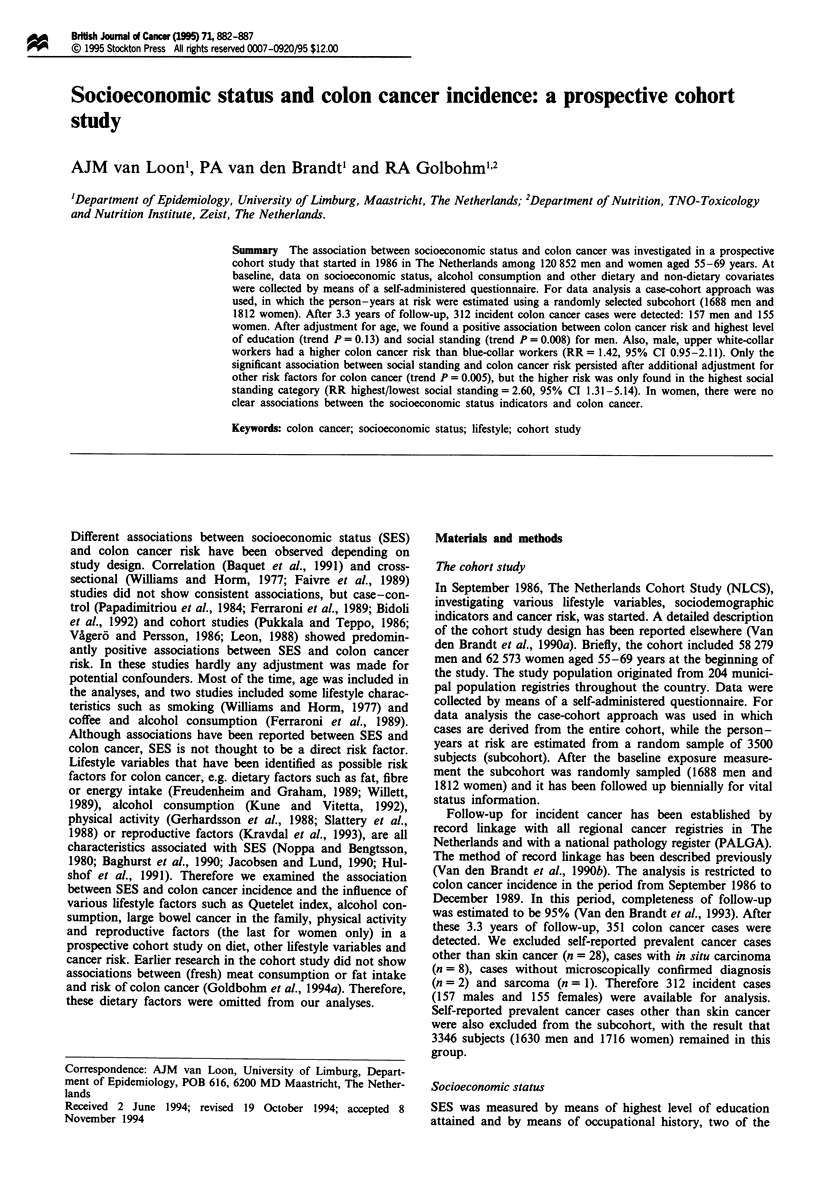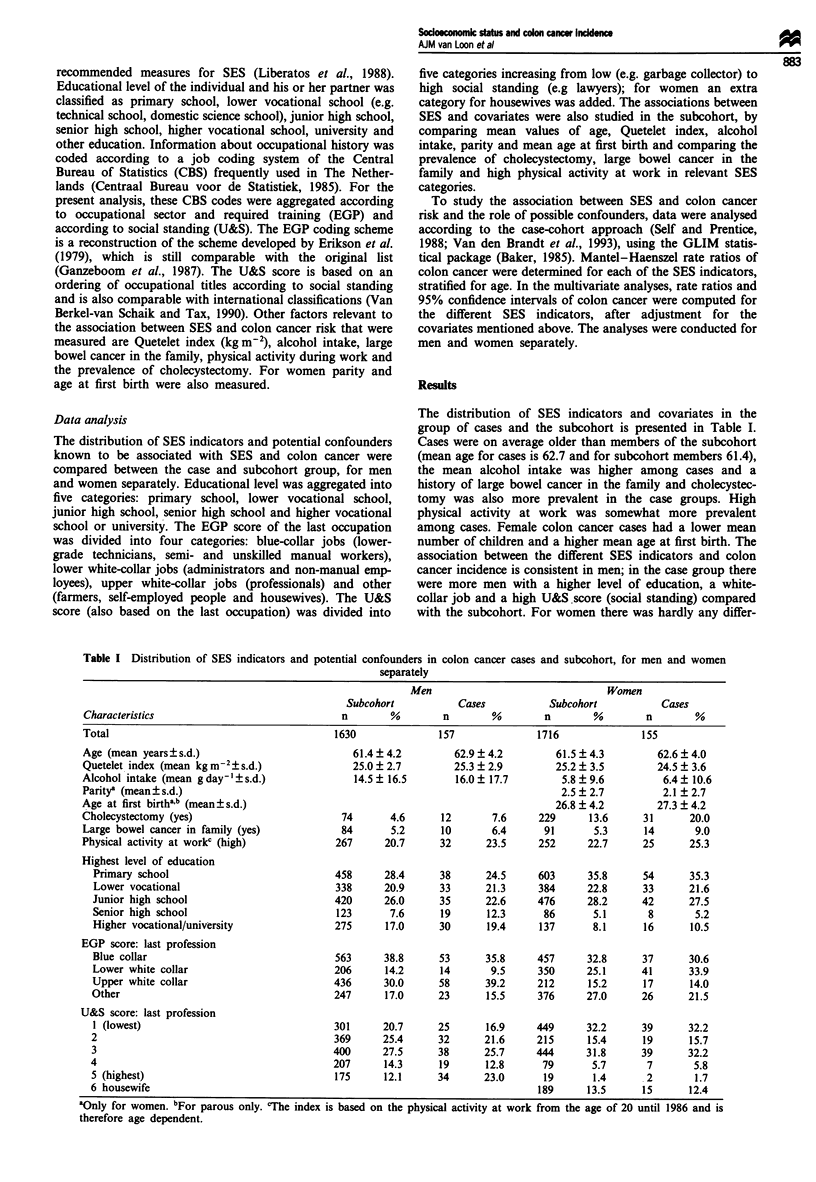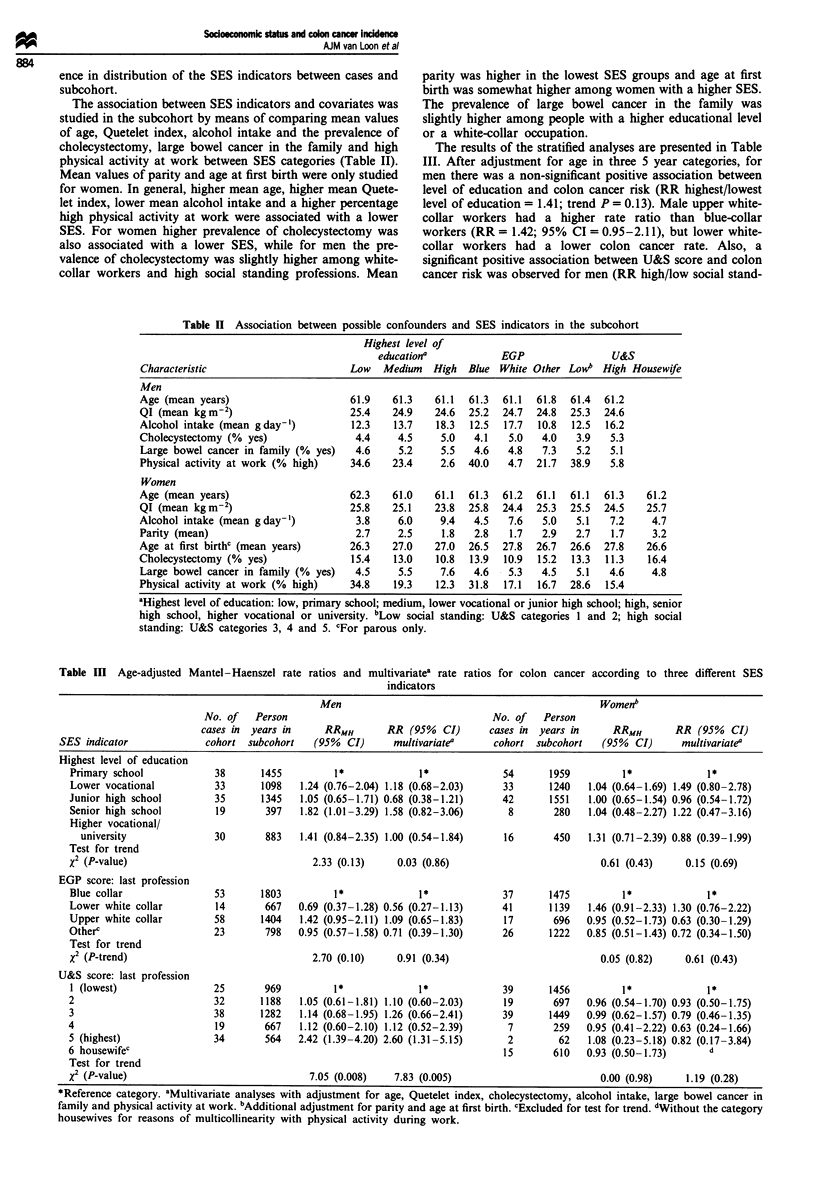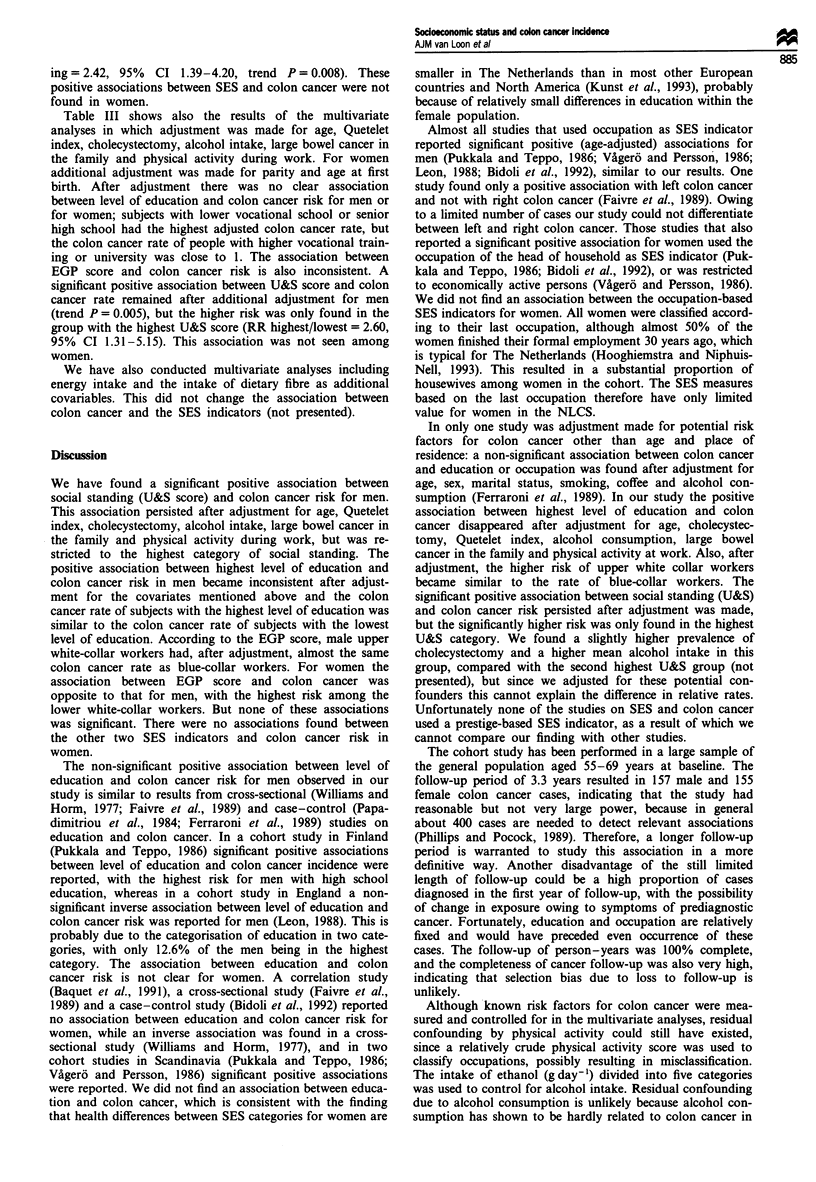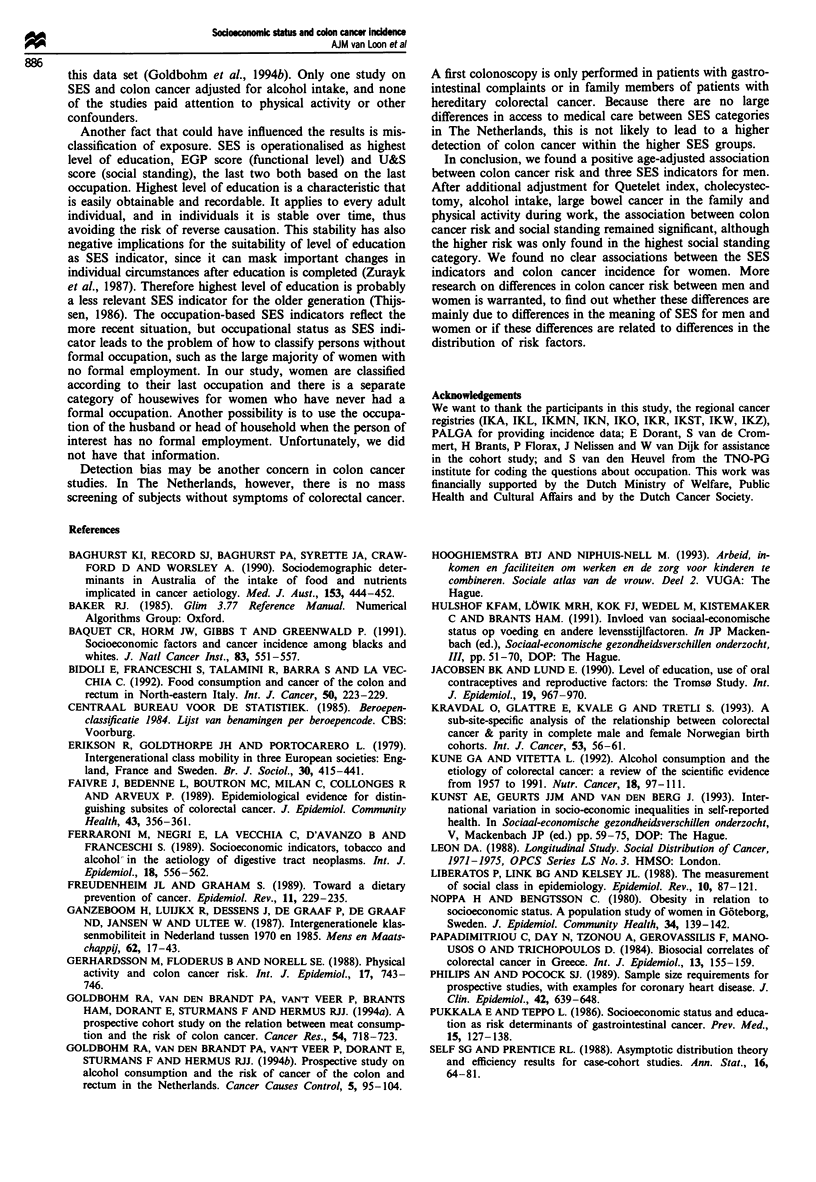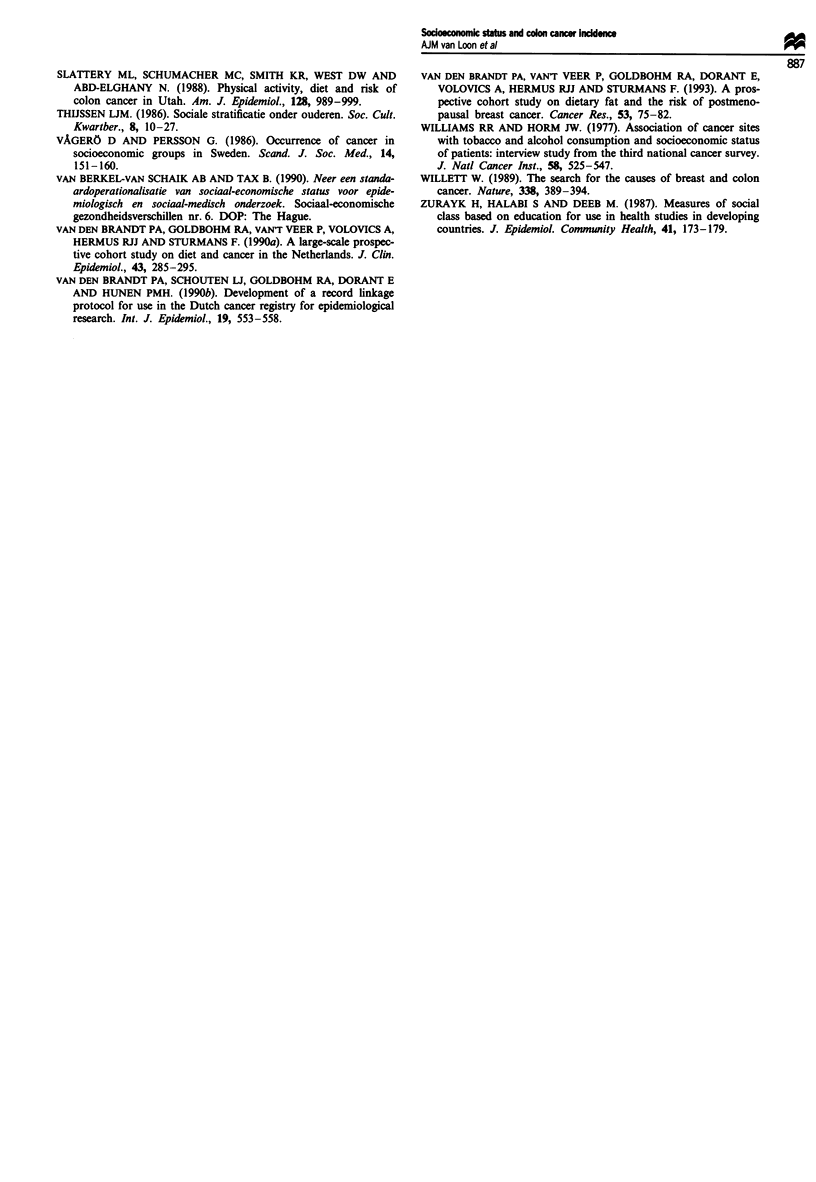# Socioeconomic status and colon cancer incidence: a prospective cohort study.

**DOI:** 10.1038/bjc.1995.170

**Published:** 1995-04

**Authors:** A. J. van Loon, P. A. van den Brandt, R. A. Golbohm

**Affiliations:** Department of Epidemiology, University of Limburg, Maastricht, The Netherlands.

## Abstract

The association between socioeconomic status and colon cancer was investigated in a prospective cohort study that started in 1986 in The Netherlands among 120,852 men and women aged 55-69 years. At baseline, data on socioeconomic status, alcohol consumption and other dietary and non-dietary covariates were collected by means of a self-administered questionnaire. For data analysis a case-cohort approach was used, in which the person-years at risk were estimated using a randomly selected subcohort (1688 men and 1812 women). After 3.3 years of follow-up, 312 incident colon cancer cases were detected: 157 men and 155 women. After adjustment for age, we found a positive association between colon cancer risk and highest level of education (trend P = 0.13) and social standing (trend P = 0.008) for men. Also, male, upper white-collar workers had a higher colon cancer risk than blue-collar workers (RR = 1.42, 95% CI 0.95-2.11). Only the significant association between social standing and colon cancer risk persisted after additional adjustment for other risk factors for colon cancer (trend P = 0.005), but the higher risk was only found in the highest social standing category (RR highest/lowest social standing = 2.60, 95% CI 1.31-5.14). In women, there were no clear associations between the socioeconomic status indicators and colon cancer.


British Journal of Cancer (1995) 71, 882-887
© 1995 Stockton Press All rights reserved 0007-0920/95 $12.00

### Socioeconomic status and colon cancer incidence: a prospective cohort study

AJM van Loon[1], PA van den Brandt[1] and RA Golbohm[1,2]

[1]*Department of Epidemiology, University of Limburg, Maastricht, The Netherlands;* [2]*Department of Nutrition, TNO-Toxicology and Nutrition Institute, Zeist, The Netherlands.*

**Summary** The association between socioeconomic status and colon cancer was investigated in a prospective cohort study that started in 1986 in The Netherlands among 120 852 men and women aged 55-69 years. At baseline, data on socioeconomic status, alcohol consumption and other dietary and non-dietary covariates were collected by means of a self-administered questionnaire. For data analysis a case-cohort approach was used, in which the person-years at risk were estimated using a randomly selected subcohort (1688 men and 1812 women). After 3.3 years of follow-up, 312 incident colon cancer cases were detected: 157 men and 155 women. After adjustment for age, we found a positive association between colon cancer risk and highest level of education (trend $P = 0.13$) and social standing (trend $P = 0.008$) for men. Also, male, upper white-collar workers had a higher colon cancer risk than blue-collar workers (RR = 1.42, 95% CI 0.95-2.11). Only the significant association between social standing and colon cancer risk persisted after additional adjustment for other risk factors for colon cancer (trend $P = 0.005$), but the higher risk was only found in the highest social standing category (RR highest/lowest social standing = 2.60, 95% CI 1.31-5.14). In women, there were no clear associations between the socioeconomic status indicators and colon cancer.

**Keywords:** colon cancer; socioeconomic status; lifestyle; cohort study

Different associations between socioeconomic status (SES) and colon cancer risk have been observed depending on study design. Correlation (Baquet *et al.*, 1991) and cross-sectional (Williams and Horm, 1977; Faivre *et al.*, 1989) studies did not show consistent associations, but case-control (Papadimitriou *et al.*, 1984; Ferraroni *et al.*, 1989; Bidoli *et al.*, 1992) and cohort studies (Pukkala and Teppo, 1986; Vågerö and Persson, 1986; Leon, 1988) showed predominantly positive associations between SES and colon cancer risk. In these studies hardly any adjustment was made for potential confounders. Most of the time, age was included in the analyses, and two studies included some lifestyle characteristics such as smoking (Williams and Horm, 1977) and coffee and alcohol consumption (Ferraroni *et al.*, 1989). Although associations have been reported between SES and colon cancer, SES is not thought to be a direct risk factor. Lifestyle variables that have been identified as possible risk factors for colon cancer, e.g. dietary factors such as fat, fibre or energy intake (Freudenheim and Graham, 1989; Willett, 1989), alcohol consumption (Kune and Vitetta, 1992), physical activity (Gerhardsson *et al.*, 1988; Slattery *et al.*, 1988) or reproductive factors (Kravdal *et al.*, 1993), are all characteristics associated with SES (Noppa and Bengtsson, 1980; Baghurst *et al.*, 1990; Jacobsen and Lund, 1990; Hulshof *et al.*, 1991). Therefore we examined the association between SES and colon cancer incidence and the influence of various lifestyle factors such as Quetelet index, alcohol consumption, large bowel cancer in the family, physical activity and reproductive factors (the last for women only) in a prospective cohort study on diet, other lifestyle variables and cancer risk. Earlier research in the cohort study did not show associations between (fresh) meat consumption or fat intake and risk of colon cancer (Goldbohm *et al.*, 1994*a*). Therefore, these dietary factors were omitted from our analyses.

Correspondence: AJM van Loon, University of Limburg, Department of Epidemiology, POB 616, 6200 MD Maastricht, The Netherlands
Received 2 June 1994; revised 19 October 1994; accepted 8 November 1994

### Materials and methods

### *The cohort study*

In September 1986, The Netherlands Cohort Study (NLCS), investigating various lifestyle variables, sociodemographic indicators and cancer risk, was started. A detailed description of the cohort study design has been reported elsewhere (Van den Brandt *et al.*, 1990*a*). Briefly, the cohort included 58 279 men and 62 573 women aged 55-69 years at the beginning of the study. The study population originated from 204 municipal population registries throughout the country. Data were collected by means of a self-administered questionnaire. For data analysis the case-cohort approach was used in which cases are derived from the entire cohort, while the person-years at risk are estimated from a random sample of 3500 subjects (subcohort). After the baseline exposure measurement the subcohort was randomly sampled (1688 men and 1812 women) and it has been followed up biennially for vital status information.

Follow-up for incident cancer has been established by record linkage with all regional cancer registries in The Netherlands and with a national pathology register (PALGA). The method of record linkage has been described previously (Van den Brandt *et al.*, 1990*b*). The analysis is restricted to colon cancer incidence in the period from September 1986 to December 1989. In this period, completeness of follow-up was estimated to be 95% (Van den Brandt *et al.*, 1993). After these 3.3 years of follow-up, 351 colon cancer cases were detected. We excluded self-reported prevalent cancer cases other than skin cancer ($n = 28$), cases with *in situ* carcinoma ($n = 8$), cases without microscopically confirmed diagnosis ($n = 2$) and sarcoma ($n = 1$). Therefore 312 incident cases (157 males and 155 females) were available for analysis. Self-reported prevalent cancer cases other than skin cancer were also excluded from the subcohort, with the result that 3346 subjects (1630 men and 1716 women) remained in this group.

### *Socioeconomic status*

SES was measured by means of highest level of education attained and by means of occupational history, two of the recommended measures for SES (Liberatos *et al.*, 1988). Educational level of the individual and his or her partner was classified as primary school, lower vocational school (e.g. technical school, domestic science school), junior high school, senior high school, higher vocational school, university and other education. Information about occupational history was coded according to a job coding system of the Central Bureau of Statistics (CBS) frequently used in The Netherlands (Centraal Bureau voor de Statistiek, 1985). For the present analysis, these CBS codes were aggregated according to occupational sector and required training (EGP) and according to social standing (U&S). The EGP coding scheme is a reconstruction of the scheme developed by Erikson *et al.* (1979), which is still comparable with the original list (Ganzeboom *et al.*, 1987). The U&S score is based on an ordering of occupational titles according to social standing and is also comparable with international classifications (Van Berkel-van Schaik and Tax, 1990). Other factors relevant to the association between SES and colon cancer risk that were measured are Quetelet index ($kg\,m^{-2}$), alcohol intake, large bowel cancer in the family, physical activity during work and the prevalence of cholecystectomy. For women parity and age at first birth were also measured.

### *Data analysis*

The distribution of SES indicators and potential confounders known to be associated with SES and colon cancer were compared between the case and subcohort group, for men and women separately. Educational level was aggregated into five categories: primary school, lower vocational school, junior high school, senior high school and higher vocational school or university. The EGP score of the last occupation was divided into four categories: blue-collar jobs (lower-grade technicians, semi- and unskilled manual workers), lower white-collar jobs (administrators and non-manual employees), upper white-collar jobs (professionals) and other (farmers, self-employed people and housewives). The U&S score (also based on the last occupation) was divided into five categories increasing from low (e.g. garbage collector) to high social standing (e.g lawyers); for women an extra category for housewives was added. The associations between SES and covariates were also studied in the subcohort, by comparing mean values of age, Quetelet index, alcohol intake, parity and mean age at first birth and comparing the prevalence of cholecystectomy, large bowel cancer in the family and high physical activity at work in relevant SES categories.

To study the association between SES and colon cancer risk and the role of possible confounders, data were analysed according to the case-cohort approach (Self and Prentice, 1988; Van den Brandt *et al.*, 1993), using the GLIM statistical package (Baker, 1985). Mantel–Haenszel rate ratios of colon cancer were determined for each of the SES indicators, stratified for age. In the multivariate analyses, rate ratios and 95% confidence intervals of colon cancer were computed for the different SES indicators, after adjustment for the covariates mentioned above. The analyses were conducted for men and women separately.

### Results

The distribution of SES indicators and covariates in the group of cases and the subcohort is presented in Table I. Cases were on average older than members of the subcohort (mean age for cases is 62.7 and for subcohort members 61.4), the mean alcohol intake was higher among cases and a history of large bowel cancer in the family and cholecystectomy was also more prevalent in the case groups. High physical activity at work was somewhat more prevalent among cases. Female colon cancer cases had a lower mean number of children and a higher mean age at first birth. The association between the different SES indicators and colon cancer incidence is consistent in men; in the case group there were more men with a higher level of education, a white-collar job and a high U&S score (social standing) compared with the subcohort. For women there was hardly any differ-

**Table I** Distribution of SES indicators and potential confounders in colon cancer cases and subcohort, for men and women separately

| | *Men* | | | | *Women* | | | |
|---|---|---|---|---|---|---|---|---|
| | *Subcohort* | | *Cases* | | *Subcohort* | | *Cases* | |
| *Characteristics* | n | % | n | % | n | % | n | % |
| Total | 1630 | | 157 | | 1716 | | 155 | |
| Age (mean years ± s.d.) | 61.4 ± 4.2 | | 62.9 ± 4.2 | | 61.5 ± 4.3 | | 62.6 ± 4.0 | |
| Quetelet index (mean $kg\,m^{-2}$ ± s.d.) | 25.0 ± 2.7 | | 25.3 ± 2.9 | | 25.2 ± 3.5 | | 24.5 ± 3.6 | |
| Alcohol intake (mean $g\,day^{-1}$ ± s.d.) | 14.5 ± 16.5 | | 16.0 ± 17.7 | | 5.8 ± 9.6 | | 6.4 ± 10.6 | |
| Parity[a] (mean ± s.d.) | | | | | 2.5 ± 2.7 | | 2.1 ± 2.7 | |
| Age at first birth[a,b] (mean ± s.d.) | | | | | 26.8 ± 4.2 | | 27.3 ± 4.2 | |
| Cholecystectomy (yes) | 74 | 4.6 | 12 | 7.6 | 229 | 13.6 | 31 | 20.0 |
| Large bowel cancer in family (yes) | 84 | 5.2 | 10 | 6.4 | 91 | 5.3 | 14 | 9.0 |
| Physical activity at work[c] (high) | 267 | 20.7 | 32 | 23.5 | 252 | 22.7 | 25 | 25.3 |
| Highest level of education | | | | | | | | |
| Primary school | 458 | 28.4 | 38 | 24.5 | 603 | 35.8 | 54 | 35.3 |
| Lower vocational | 338 | 20.9 | 33 | 21.3 | 384 | 22.8 | 33 | 21.6 |
| Junior high school | 420 | 26.0 | 35 | 22.6 | 476 | 28.2 | 42 | 27.5 |
| Senior high school | 123 | 7.6 | 19 | 12.3 | 86 | 5.1 | 8 | 5.2 |
| Higher vocational/university | 275 | 17.0 | 30 | 19.4 | 137 | 8.1 | 16 | 10.5 |
| EGP score: last profession | | | | | | | | |
| Blue collar | 563 | 38.8 | 53 | 35.8 | 457 | 32.8 | 37 | 30.6 |
| Lower white collar | 206 | 14.2 | 14 | 9.5 | 350 | 25.1 | 41 | 33.9 |
| Upper white collar | 436 | 30.0 | 58 | 39.2 | 212 | 15.2 | 17 | 14.0 |
| Other | 247 | 17.0 | 23 | 15.5 | 376 | 27.0 | 26 | 21.5 |
| U&S score: last profession | | | | | | | | |
| 1 (lowest) | 301 | 20.7 | 25 | 16.9 | 449 | 32.2 | 39 | 32.2 |
| 2 | 369 | 25.4 | 32 | 21.6 | 215 | 15.4 | 19 | 15.7 |
| 3 | 400 | 27.5 | 38 | 25.7 | 444 | 31.8 | 39 | 32.2 |
| 4 | 207 | 14.3 | 19 | 12.8 | 79 | 5.7 | 7 | 5.8 |
| 5 (highest) | 175 | 12.1 | 34 | 23.0 | 19 | 1.4 | 2 | 1.7 |
| 6 housewife | | | | | 189 | 13.5 | 15 | 12.4 |

[a]Only for women. [b]For parous only. [c]The index is based on the physical activity at work from the age of 20 until 1986 and is therefore age dependent.

ence in distribution of the SES indicators between cases and subcohort.

The association between SES indicators and covariates was studied in the subcohort by means of comparing mean values of age, Quetelet index, alcohol intake and the prevalence of cholecystectomy, large bowel cancer in the family and high physical activity at work between SES categories (Table II). Mean values of parity and age at first birth were only studied for women. In general, higher mean age, higher mean Quetelet index, lower mean alcohol intake and a higher percentage high physical activity at work were associated with a lower SES. For women higher prevalence of cholecystectomy was also associated with a lower SES, while for men the prevalence of cholecystectomy was slightly higher among white-collar workers and high social standing professions. Mean parity was higher in the lowest SES groups and age at first birth was somewhat higher among women with a higher SES. The prevalence of large bowel cancer in the family was slightly higher among people with a higher educational level or a white-collar occupation.

The results of the stratified analyses are presented in Table III. After adjustment for age in three 5 year categories, for men there was a non-significant positive association between level of education and colon cancer risk (RR highest/lowest level of education = 1.41; trend $P = 0.13$). Male upper white-collar workers had a higher rate ratio than blue-collar workers (RR = 1.42; 95% CI = 0.95–2.11), but lower white-collar workers had a lower colon cancer rate. Also, a significant positive association between U&S score and colon cancer risk was observed for men (RR high/low social stand-

**Table II** Association between possible confounders and SES indicators in the subcohort

| *Characteristic* | *Highest level of education*[a] *Low* | *Medium* | *High* | *EGP* *Blue* | *White* | *Other* | *U&S* *Low*[b] | *High* | *Housewife* |
|---|---|---|---|---|---|---|---|---|---|
| *Men* | | | | | | | | | |
| Age (mean years) | 61.9 | 61.3 | 61.1 | 61.3 | 61.1 | 61.8 | 61.4 | 61.2 | |
| QI (mean kg m$^{-2}$) | 25.4 | 24.9 | 24.6 | 25.2 | 24.7 | 24.8 | 25.3 | 24.6 | |
| Alcohol intake (mean g day$^{-1}$) | 12.3 | 13.7 | 18.3 | 12.5 | 17.7 | 10.8 | 12.5 | 16.2 | |
| Cholecystectomy (% yes) | 4.4 | 4.5 | 5.0 | 4.1 | 5.0 | 4.0 | 3.9 | 5.3 | |
| Large bowel cancer in family (% yes) | 4.6 | 5.2 | 5.5 | 4.6 | 4.8 | 7.3 | 5.2 | 5.1 | |
| Physical activity at work (% high) | 34.6 | 23.4 | 2.6 | 40.0 | 4.7 | 21.7 | 38.9 | 5.8 | |
| *Women* | | | | | | | | | |
| Age (mean years) | 62.3 | 61.0 | 61.1 | 61.3 | 61.2 | 61.1 | 61.1 | 61.3 | 61.2 |
| QI (mean kg m$^{-2}$) | 25.8 | 25.1 | 23.8 | 25.8 | 24.4 | 25.3 | 25.5 | 24.5 | 25.7 |
| Alcohol intake (mean g day$^{-1}$) | 3.8 | 6.0 | 9.4 | 4.5 | 7.6 | 5.0 | 5.1 | 7.2 | 4.7 |
| Parity (mean) | 2.7 | 2.5 | 1.8 | 2.8 | 1.7 | 2.9 | 2.7 | 1.7 | 3.2 |
| Age at first birth[c] (mean years) | 26.3 | 27.0 | 27.0 | 26.5 | 27.8 | 26.7 | 26.6 | 27.8 | 26.6 |
| Cholecystectomy (% yes) | 15.4 | 13.0 | 10.8 | 13.9 | 10.9 | 15.2 | 13.3 | 11.3 | 16.4 |
| Large bowel cancer in family (% yes) | 4.5 | 5.5 | 7.6 | 4.6 | 5.3 | 4.5 | 5.1 | 4.6 | 4.8 |
| Physical activity at work (% high) | 34.8 | 19.3 | 12.3 | 31.8 | 17.1 | 16.7 | 28.6 | 15.4 | |

[a]Highest level of education: low, primary school; medium, lower vocational or junior high school; high, senior high school, higher vocational or university. [b]Low social standing: U&S categories 1 and 2; high social standing: U&S categories 3, 4 and 5. [c]For parous only.

**Table III** Age-adjusted Mantel–Haenszel rate ratios and multivariate[a] rate ratios for colon cancer according to three different SES indicators

| *SES indicator* | *Men* *No. of cases in cohort* | *Person years in subcohort* | *$RR_{MH}$ (95% CI)* | *RR (95% CI) multivariate*[a] | *Women*[b] *No. of cases in cohort* | *Person years in subcohort* | *$RR_{MH}$ (95% CI)* | *RR (95% CI) multivariate*[a] |
|---|---|---|---|---|---|---|---|---|
| Highest level of education | | | | | | | | |
| Primary school | 38 | 1455 | 1* | 1* | 54 | 1959 | 1* | 1* |
| Lower vocational | 33 | 1098 | 1.24 (0.76–2.04) | 1.18 (0.68–2.03) | 33 | 1240 | 1.04 (0.64–1.69) | 1.49 (0.80–2.78) |
| Junior high school | 35 | 1345 | 1.05 (0.65–1.71) | 0.68 (0.38–1.21) | 42 | 1551 | 1.00 (0.65–1.54) | 0.96 (0.54–1.72) |
| Senior high school | 19 | 397 | 1.82 (1.01–3.29) | 1.58 (0.82–3.06) | 8 | 280 | 1.04 (0.48–2.27) | 1.22 (0.47–3.16) |
| Higher vocational/ university | 30 | 883 | 1.41 (0.84–2.35) | 1.00 (0.54–1.84) | 16 | 450 | 1.31 (0.71–2.39) | 0.88 (0.39–1.99) |
| Test for trend $\chi^2$ (*P*-value) | | | 2.33 (0.13) | 0.03 (0.86) | | | 0.61 (0.43) | 0.15 (0.69) |
| EGP score: last profession | | | | | | | | |
| Blue collar | 53 | 1803 | 1* | 1* | 37 | 1475 | 1* | 1* |
| Lower white collar | 14 | 667 | 0.69 (0.37–1.28) | 0.56 (0.27–1.13) | 41 | 1139 | 1.46 (0.91–2.33) | 1.30 (0.76–2.22) |
| Upper white collar | 58 | 1404 | 1.42 (0.95–2.11) | 1.09 (0.65–1.83) | 17 | 696 | 0.95 (0.52–1.73) | 0.63 (0.30–1.29) |
| Other[c] | 23 | 798 | 0.95 (0.57–1.58) | 0.71 (0.39–1.30) | 26 | 1222 | 0.85 (0.51–1.43) | 0.72 (0.34–1.50) |
| Test for trend $\chi^2$ (*P*-trend) | | | 2.70 (0.10) | 0.91 (0.34) | | | 0.05 (0.82) | 0.61 (0.43) |
| U&S score: last profession | | | | | | | | |
| 1 (lowest) | 25 | 969 | 1* | 1* | 39 | 1456 | 1* | 1* |
| 2 | 32 | 1188 | 1.05 (0.61–1.81) | 1.10 (0.60–2.03) | 19 | 697 | 0.96 (0.54–1.70) | 0.93 (0.50–1.75) |
| 3 | 38 | 1282 | 1.14 (0.68–1.95) | 1.26 (0.66–2.41) | 39 | 1449 | 0.99 (0.62–1.57) | 0.79 (0.46–1.35) |
| 4 | 19 | 667 | 1.12 (0.60–2.10) | 1.12 (0.52–2.39) | 7 | 259 | 0.95 (0.41–2.22) | 0.63 (0.24–1.66) |
| 5 (highest) | 34 | 564 | 2.42 (1.39–4.20) | 2.60 (1.31–5.15) | 2 | 62 | 1.08 (0.23–5.18) | 0.82 (0.17–3.84) |
| 6 housewife[c] | | | | | 15 | 610 | 0.93 (0.50–1.73) | [d] |
| Test for trend $\chi^2$ (*P*-value) | | | 7.05 (0.008) | 7.83 (0.005) | | | 0.00 (0.98) | 1.19 (0.28) |

*Reference category. [a]Multivariate analyses with adjustment for age, Quetelet index, cholecystectomy, alcohol intake, large bowel cancer in family and physical activity at work. [b]Additional adjustment for parity and age at first birth. [c]Excluded for test for trend. [d]Without the category housewives for reasons of multicollinearity with physical activity during work.

ing = 2.42, 95% CI 1.39–4.20, trend $P = 0.008$). These positive associations between SES and colon cancer were not found in women.

Table III shows also the results of the multivariate analyses in which adjustment was made for age, Quetelet index, cholecystectomy, alcohol intake, large bowel cancer in the family and physical activity during work. For women additional adjustment was made for parity and age at first birth. After adjustment there was no clear association between level of education and colon cancer risk for men or for women; subjects with lower vocational school or senior high school had the highest adjusted colon cancer rate, but the colon cancer rate of people with higher vocational training or university was close to 1. The association between EGP score and colon cancer risk is also inconsistent. A significant positive association between U&S score and colon cancer rate remained after additional adjustment for men (trend $P = 0.005$), but the higher risk was only found in the group with the highest U&S score (RR highest/lowest = 2.60, 95% CI 1.31–5.15). This association was not seen among women.

We have also conducted multivariate analyses including energy intake and the intake of dietary fibre as additional covariables. This did not change the association between colon cancer and the SES indicators (not presented).

### Discussion

We have found a significant positive association between social standing (U&S score) and colon cancer risk for men. This association persisted after adjustment for age, Quetelet index, cholecystectomy, alcohol intake, large bowel cancer in the family and physical activity during work, but was restricted to the highest category of social standing. The positive association between highest level of education and colon cancer risk in men became inconsistent after adjustment for the covariates mentioned above and the colon cancer rate of subjects with the highest level of education was similar to the colon cancer rate of subjects with the lowest level of education. According to the EGP score, male upper white-collar workers had, after adjustment, almost the same colon cancer rate as blue-collar workers. For women the association between EGP score and colon cancer was opposite to that for men, with the highest risk among the lower white-collar workers. But none of these associations was significant. There were no associations found between the other two SES indicators and colon cancer risk in women.

The non-significant positive association between level of education and colon cancer risk for men observed in our study is similar to results from cross-sectional (Williams and Horm, 1977; Faivre *et al.*, 1989) and case–control (Papadimitriou *et al.*, 1984; Ferraroni *et al.*, 1989) studies on education and colon cancer. In a cohort study in Finland (Pukkala and Teppo, 1986) significant positive associations between level of education and colon cancer incidence were reported, with the highest risk for men with high school education, whereas in a cohort study in England a non-significant inverse association between level of education and colon cancer risk was reported for men (Leon, 1988). This is probably due to the categorisation of education in two categories, with only 12.6% of the men being in the highest category. The association between education and colon cancer risk is not clear for women. A correlation study (Baquet *et al.*, 1991), a cross-sectional study (Faivre *et al.*, 1989) and a case–control study (Bidoli *et al.*, 1992) reported no association between education and colon cancer risk for women, while an inverse association was found in a cross-sectional study (Williams and Horm, 1977), and in two cohort studies in Scandinavia (Pukkala and Teppo, 1986; Vågerö and Persson, 1986) significant positive associations were reported. We did not find an association between education and colon cancer, which is consistent with the finding that health differences between SES categories for women are smaller in The Netherlands than in most other European countries and North America (Kunst *et al.*, 1993), probably because of relatively small differences in education within the female population.

Almost all studies that used occupation as SES indicator reported significant positive (age-adjusted) associations for men (Pukkala and Teppo, 1986; Vågerö and Persson, 1986; Leon, 1988; Bidoli *et al.*, 1992), similar to our results. One study found only a positive association with left colon cancer and not with right colon cancer (Faivre *et al.*, 1989). Owing to a limited number of cases our study could not differentiate between left and right colon cancer. Those studies that also reported a significant positive association for women used the occupation of the head of household as SES indicator (Pukkala and Teppo, 1986; Bidoli *et al.*, 1992), or was restricted to economically active persons (Vågerö and Persson, 1986). We did not find an association between the occupation-based SES indicators for women. All women were classified according to their last occupation, although almost 50% of the women finished their formal employment 30 years ago, which is typical for The Netherlands (Hooghiemstra and Niphuis-Nell, 1993). This resulted in a substantial proportion of housewives among women in the cohort. The SES measures based on the last occupation therefore have only limited value for women in the NLCS.

In only one study was adjustment made for potential risk factors for colon cancer other than age and place of residence: a non-significant association between colon cancer and education or occupation was found after adjustment for age, sex, marital status, smoking, coffee and alcohol consumption (Ferraroni *et al.*, 1989). In our study the positive association between highest level of education and colon cancer disappeared after adjustment for age, cholecystectomy, Quetelet index, alcohol consumption, large bowel cancer in the family and physical activity at work. Also, after adjustment, the higher risk of upper white collar workers became similar to the rate of blue-collar workers. The significant positive association between social standing (U&S) and colon cancer risk persisted after adjustment was made, but the significantly higher risk was only found in the highest U&S category. We found a slightly higher prevalence of cholecystectomy and a higher mean alcohol intake in this group, compared with the second highest U&S group (not presented), but since we adjusted for these potential confounders this cannot explain the difference in relative rates. Unfortunately none of the studies on SES and colon cancer used a prestige-based SES indicator, as a result of which we cannot compare our finding with other studies.

The cohort study has been performed in a large sample of the general population aged 55–69 years at baseline. The follow-up period of 3.3 years resulted in 157 male and 155 female colon cancer cases, indicating that the study had reasonable but not very large power, because in general about 400 cases are needed to detect relevant associations (Phillips and Pocock, 1989). Therefore, a longer follow-up period is warranted to study this association in a more definitive way. Another disadvantage of the still limited length of follow-up could be a high proportion of cases diagnosed in the first year of follow-up, with the possibility of change in exposure owing to symptoms of prediagnostic cancer. Fortunately, education and occupation are relatively fixed and would have preceded even occurrence of these cases. The follow-up of person–years was 100% complete, and the completeness of cancer follow-up was also very high, indicating that selection bias due to loss to follow-up is unlikely.

Although known risk factors for colon cancer were measured and controlled for in the multivariate analyses, residual confounding by physical activity could still have existed, since a relatively crude physical activity score was used to classify occupations, possibly resulting in misclassification. The intake of ethanol (g $day^{-1}$) divided into five categories was used to control for alcohol intake. Residual confounding due to alcohol consumption is unlikely because alcohol consumption has shown to be hardly related to colon cancer in this data set (Goldbohm *et al.*, 1994*b*). Only one study on SES and colon cancer adjusted for alcohol intake, and none of the studies paid attention to physical activity or other confounders.

Another fact that could have influenced the results is misclassification of exposure. SES is operationalised as highest level of education, EGP score (functional level) and U&S score (social standing), the last two both based on the last occupation. Highest level of education is a characteristic that is easily obtainable and recordable. It applies to every adult individual, and in individuals it is stable over time, thus avoiding the risk of reverse causation. This stability has also negative implications for the suitability of level of education as SES indicator, since it can mask important changes in individual circumstances after education is completed (Zurayk *et al.*, 1987). Therefore highest level of education is probably a less relevant SES indicator for the older generation (Thijssen, 1986). The occupation-based SES indicators reflect the more recent situation, but occupational status as SES indicator leads to the problem of how to classify persons without formal occupation, such as the large majority of women with no formal employment. In our study, women are classified according to their last occupation and there is a separate category of housewives for women who have never had a formal occupation. Another possibility is to use the occupation of the husband or head of household when the person of interest has no formal employment. Unfortunately, we did not have that information.

Detection bias may be another concern in colon cancer studies. In The Netherlands, however, there is no mass screening of subjects without symptoms of colorectal cancer. A first colonoscopy is only performed in patients with gastrointestinal complaints or in family members of patients with hereditary colorectal cancer. Because there are no large differences in access to medical care between SES categories in The Netherlands, this is not likely to lead to a higher detection of colon cancer within the higher SES groups.

In conclusion, we found a positive age-adjusted association between colon cancer risk and three SES indicators for men. After additional adjustment for Quetelet index, cholecystectomy, alcohol intake, large bowel cancer in the family and physical activity during work, the association between colon cancer risk and social standing remained significant, although the higher risk was only found in the highest social standing category. We found no clear associations between the SES indicators and colon cancer incidence for women. More research on differences in colon cancer risk between men and women is warranted, to find out whether these differences are mainly due to differences in the meaning of SES for men and women or if these differences are related to differences in the distribution of risk factors.

**Acknowledgements**

We want to thank the participants in this study, the regional cancer registries (IKA, IKL, IKMN, IKN, IKO, IKR, IKST, IKW, IKZ), PALGA for providing incidence data; E Dorant, S van de Crommert, H Brants, P Florax, J Nelissen and W van Dijk for assistance in the cohort study; and S van den Heuvel from the TNO-PG institute for coding the questions about occupation. This work was financially supported by the Dutch Ministry of Welfare, Public Health and Cultural Affairs and by the Dutch Cancer Society.

**References**

BAGHURST KI, RECORD SJ, BAGHURST PA, SYRETTE JA, CRAWFORD D AND WORSLEY A. (1990). Sociodemographic determinants in Australia of the intake of food and nutrients implicated in cancer aetiology. *Med. J. Aust.*, **153**, 444–452.

BAKER RJ. (1985). *Glim 3.77 Reference Manual.* Numerical Algorithms Group: Oxford.

BAQUET CR, HORM JW, GIBBS T AND GREENWALD P. (1991). Socioeconomic factors and cancer incidence among blacks and whites. *J. Natl Cancer Inst.*, **83**, 551–557.

BIDOLI E, FRANCESCHI S, TALAMINI R, BARRA S AND LA VECCHIA C. (1992). Food consumption and cancer of the colon and rectum in North-eastern Italy. *Int. J. Cancer*, **50**, 223–229.

CENTRAAL BUREAU VOOR DE STATISTIEK. (1985). *Beroepenclassificatie 1984. Lijst van benamingen per beroepencode.* CBS: Voorburg.

ERIKSON R, GOLDTHORPE JH AND PORTOCARERO L. (1979). Intergenerational class mobility in three European societies: England, France and Sweden. *Br. J. Sociol.*, **30**, 415–441.

FAIVRE J, BEDENNE L, BOUTRON MC, MILAN C, COLLONGES R AND ARVEUX P. (1989). Epidemiological evidence for distinguishing subsites of colorectal cancer. *J. Epidemiol. Community Health*, **43**, 356–361.

FERRARONI M, NEGRI E, LA VECCHIA C, D'AVANZO B AND FRANCESCHI S. (1989). Socioeconomic indicators, tobacco and alcohol in the aetiology of digestive tract neoplasms. *Int. J. Epidemiol.*, **18**, 556–562.

FREUDENHEIM JL AND GRAHAM S. (1989). Toward a dietary prevention of cancer. *Epidemiol. Rev.*, **11**, 229–235.

GANZEBOOM H, LUIJKX R, DESSENS J, DE GRAAF P, DE GRAAF ND, JANSEN W AND ULTEE W. (1987). Intergenerationele klassenmobiliteit in Nederland tussen 1970 en 1985. *Mens en Maatschappij*, **62**, 17–43.

GERHARDSSON M, FLODERUS B AND NORELL SE. (1988). Physical activity and colon cancer risk. *Int. J. Epidemiol.*, **17**, 743–746.

GOLDBOHM RA, VAN DEN BRANDT PA, VAN'T VEER P, BRANTS HAM, DORANT E, STURMANS F AND HERMUS RJJ. (1994*a*). A prospective cohort study on the relation between meat consumption and the risk of colon cancer. *Cancer Res.*, **54**, 718–723.

GOLDBOHM RA, VAN DEN BRANDT PA, VAN'T VEER P, DORANT E, STURMANS F AND HERMUS RJJ. (1994*b*). Prospective study on alcohol consumption and the risk of cancer of the colon and rectum in the Netherlands. *Cancer Causes Control*, **5**, 95–104.

HOOGHIEMSTRA BTJ AND NIPHUIS-NELL M. (1993). *Arbeid, inkomen en faciliteiten om werken en de zorg voor kinderen te combineren. Sociale atlas van de vrouw. Deel 2.* VUGA: The Hague.

HULSHOF KFAM, LÖWIK MRH, KOK FJ, WEDEL M, KISTEMAKER C AND BRANTS HAM. (1991). Invloed van sociaal-economische status op voeding en andere levensstijlfactoren. *In* JP Mackenbach (ed.), *Sociaal-economische gezondheidsverschillen onderzocht, III*, pp. 51–70, DOP: The Hague.

JACOBSEN BK AND LUND E. (1990). Level of education, use of oral contraceptives and reproductive factors: the Tromsø Study. *Int. J. Epidemiol.*, **19**, 967–970.

KRAVDAL O, GLATTRE E, KVALE G AND TRETLI S. (1993). A sub-site-specific analysis of the relationship between colorectal cancer & parity in complete male and female Norwegian birth cohorts. *Int. J. Cancer*, **53**, 56–61.

KUNE GA AND VITETTA L. (1992). Alcohol consumption and the etiology of colorectal cancer: a review of the scientific evidence from 1957 to 1991. *Nutr. Cancer*, **18**, 97–111.

KUNST AE, GEURTS JJM AND VAN DEN BERG J. (1993). International variation in socio-economic inequalities in self-reported health. In *Sociaal-economische gezondheidsverschillen onderzocht*, V, Mackenbach JP (ed.) pp. 59–75, DOP: The Hague.

LEON DA. (1988). *Longitudinal Study. Social Distribution of Cancer, 1971–1975, OPCS Series LS No. 3.* HMSO: London.

LIBERATOS P, LINK BG AND KELSEY JL. (1988). The measurement of social class in epidemiology. *Epidemiol. Rev.*, **10**, 87–121.

NOPPA H AND BENGTSSON C. (1980). Obesity in relation to socioeconomic status. A population study of women in Göteborg, Sweden. *J. Epidemiol. Community Health*, **34**, 139–142.

PAPADIMITRIOU C, DAY N, TZONOU A, GEROVASSILIS F, MANOUSOS O AND TRICHOPOULOS D. (1984). Biosocial correlates of colorectal cancer in Greece. *Int. J. Epidemiol.*, **13**, 155–159.

PHILIPS AN AND POCOCK SJ. (1989). Sample size requirements for prospective studies, with examples for coronary heart disease. *J. Clin. Epidemiol.*, **42**, 639–648.

PUKKALA E AND TEPPO L. (1986). Socioeconomic status and education as risk determinants of gastrointestinal cancer. *Prev. Med.*, **15**, 127–138.

SELF SG AND PRENTICE RL. (1988). Asymptotic distribution theory and efficiency results for case-cohort studies. *Ann. Stat.*, **16**, 64–81.

SLATTERY ML, SCHUMACHER MC, SMITH KR, WEST DW AND ABD-ELGHANY N. (1988). Physical activity, diet and risk of colon cancer in Utah. *Am. J. Epidemiol.*, **128,** 989–999.

THIJSSEN LJM. (1986). Sociale stratificatie onder ouderen. *Soc. Cult. Kwartber.*, **8,** 10–27.

VÅGERÖ D AND PERSSON G. (1986). Occurrence of cancer in socioeconomic groups in Sweden. *Scand. J. Soc. Med.*, **14,** 151–160.

VAN BERKEL-VAN SCHAIK AB AND TAX B. (1990). *Neer een standaardoperationalisatie van sociaal-economische status voor epidemiologisch en sociaal-medisch onderzoek.* Sociaal-economische gezondheidsverschillen nr. 6. DOP: The Hague.

VAN DEN BRANDT PA, GOLDBOHM RA, VAN'T VEER P, VOLOVICS A, HERMUS RJJ AND STURMANS F. (1990*a*). A large-scale prospective cohort study on diet and cancer in the Netherlands. *J. Clin. Epidemiol.*, **43,** 285–295.

VAN DEN BRANDT PA, SCHOUTEN LJ, GOLDBOHM RA, DORANT E AND HUNEN PMH. (1990*b*). Development of a record linkage protocol for use in the Dutch cancer registry for epidemiological research. *Int. J. Epidemiol.*, **19,** 553–558.

VAN DEN BRANDT PA, VAN'T VEER P, GOLDBOHM RA, DORANT E, VOLOVICS A, HERMUS RJJ AND STURMANS F. (1993). A prospective cohort study on dietary fat and the risk of postmenopausal breast cancer. *Cancer Res.*, **53,** 75–82.

WILLIAMS RR AND HORM JW. (1977). Association of cancer sites with tobacco and alcohol consumption and socioeconomic status of patients: interview study from the third national cancer survey. *J. Natl Cancer Inst.*, **58,** 525–547.

WILLETT W. (1989). The search for the causes of breast and colon cancer. *Nature*, **338,** 389–394.

ZURAYK H, HALABI S AND DEEB M. (1987). Measures of social class based on education for use in health studies in developing countries. *J. Epidemiol. Community Health*, **41,** 173–179.